# Overall survival and adverse events after treatment with darolutamide vs. apalutamide vs. enzalutamide for high-risk non-metastatic castration-resistant prostate cancer: a systematic review and network meta-analysis

**DOI:** 10.1038/s41391-021-00395-4

**Published:** 2021-05-30

**Authors:** Mike Wenzel, Luigi Nocera, Claudia Collà Ruvolo, Christoph Würnschimmel, Zhe Tian, Shahrokh F. Shariat, Fred Saad, Derya Tilki, Markus Graefen, Luis A. Kluth, Alberto Briganti, Philipp Mandel, Francesco Montorsi, Felix K. H. Chun, Pierre I. Karakiewicz

**Affiliations:** 1 Department of Urology, Goethe University Hospital Frankfurt, Frankfurt am Main, Germany; 2 Division of Urology, Cancer Prognostics and Health Outcomes Unit, University of Montréal Health Center, Montréal, QC Canada; 3 Department of Urology and Division of Experimental Oncology, URI, Urological Research Institute, IRCCS San Raffaele Scientific Institute, Milan, Italy; 4 Department of Neurosciences, Reproductive Sciences and Odontostomatology, University of Naples Federico II, Naples, Italy; 5 Martini-Klinik Prostate Cancer Center, University Hospital Hamburg-Eppendorf, Hamburg, Germany; 6 Department of Urology, Comprehensive Cancer Center, Medical University of Vienna, Vienna, Austria; 7 Department of Urology, Weill Cornell Medical College, New York, NY USA; 8 Department of Urology, University of Texas Southwestern, Dallas, TX USA; 9 Department of Urology, Second Faculty of Medicine, Charles University, Prag, Czechia; 10 Institute for Urology and Reproductive Health, I.M. Sechenov First Moscow State Medical University, Moscow, Russia; 11 Division of Urology, Department of Special Surgery, Jordan University Hospital, The University of Jordan, Amman, Jordan; 12 Department of Urology, University Hospital Hamburg-Eppendorf, Hamburg, Germany

**Keywords:** Cancer therapy, Prostate cancer

## Abstract

**Background:**

The most recent overall survival (OS) and adverse event (AE) data have not been compared for the three guideline-recommended high-risk non-metastatic castration-resistant prostate cancer (nmCRPC) treatment alternatives.

**Methods:**

We performed a systematic review and network meta-analysis focusing on OS and AE according to the most recent apalutamide, enzalutamide, and darolutamide reports. We systematically examined and compared apalutamide vs. enzalutamide vs. darolutamide efficacy and toxicity, relative to ADT according to PRISMA. We relied on PubMed search for most recent reports addressing prospective randomized trials with proven predefined OS benefit, relative to ADT: SPARTAN, PROSPER, and ARAMIS. OS represented the primary outcome and AEs represented secondary outcomes.

**Results:**

Overall, data originated from 4117 observations made within the three trials that were analyzed. Regarding OS benefit relative to ADT, darolutamide ranked first, followed by enzalutamide and apalutamide, in that order. In the subgroup of PSA-doubling time (PSA-DT) ≤ 6 months patients, enzalutamide ranked first, followed by darolutamide and apalutamide in that order. Conversely, in the subgroup of PSA-DT 6–10 months patients, darolutamide ranked first, followed by apalutamide and enzalutamide, in that order. Regarding grade 3+ AEs, darolutamide was most favorable, followed by enzalutamide and apalutamide, in that order.

**Conclusion:**

The current network meta-analysis suggests the highest OS efficacy and lowest grade 3+ toxicity for darolutamide. However, in the PSA-DT ≤ 6 months subgroup, the highest efficacy was recorded for enzalutamide. It is noteworthy that study design, study population, and follow-up duration represent some of the potentially critical differences that distinguish between the three studies and remained statistically unaccounted for using the network meta-analysis methodology. Those differences should be strongly considered in the interpretation of the current and any network meta-analyses.

## Background

Based on statistical criteria, three prospective randomized controlled trials (RCT) testing apalutamide, enzalutamide, and darolutamide, have demonstrated an overall survival (OS) benefit for each of the three androgen receptor-axis-targeted therapies (ARAT), relative to androgen deprivation therapy (ADT), in high-risk non-metastatic castration-resistant prostate cancer (nmCRPC) [1–3]. Based on less mature follow-up than currently available, the findings of these three RCTs have been compared within five previous network meta-analyses (NMA) [4–12]. Of those, four addressed OS. Specifically, they relied on 18-month median follow-up for darolutamide, 20-month median follow-up for apalutamide, and up to 48-month median follow-up for enzalutamide. However, the most recent updates provide 52-month median follow-up for apalutamide, 48 median months for enzalutamide, and the 28-month median follow-up for darolutamide. These most current and most mature data have not been used to compare OS and/or adverse events (AE) related to the use of the three ARATs, relative to ADT.

We addressed this void. Specifically, we relied on the NMA methodology with a primary focus on OS and with a secondary focus on AEs. In addition to relying on more mature follow-up for apalutamide and darolutamide, we also provide subgroup analyses according to PSA-doubling time (PSA-DT): ≤6 vs. 6–10 months. We hypothesized that with longer follow-up, more robust OS and AE data may result in equally more robust NMA-based indirect comparisons.

## Methods

### Methodology

**Fig. 1:**
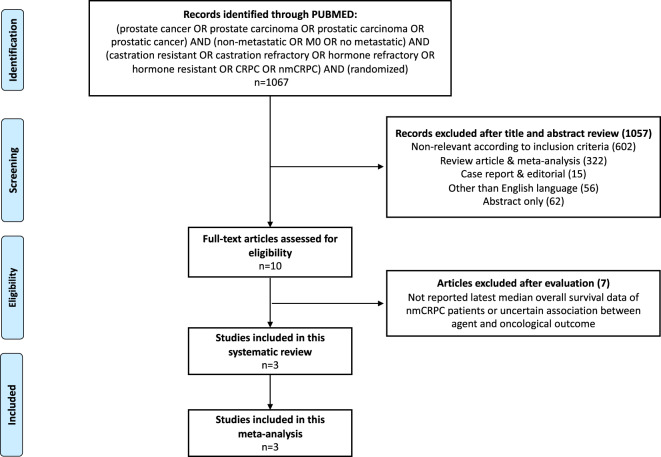
PRISMA flowchart. PRISMA (preferred reporting items for systematic reviews and meta-analyses) flow chart depicting included studies for the meta-analysis addressing overall survival and adverse events in non-metastatic castration-resistant prostate cancer (nmCRPC) patients.

We performed a systematic review and NMA of RCTs that only focused on studies, where an OS benefit was demonstrated relative to ADT alone, according to predefined statistical criteria in high-risk nmCRPC. Based on these criteria, only three studies qualified for inclusion (Fig. 1). Their most mature updates were obtained from publications [1–3]. Our study search and inclusion criteria were in accordance with the preferred reporting items for systematic reviews and meta-analyses guidelines (PRISMA) [13, 14].

### Outcome measures

The primary endpoint of this NMA was OS. Subgroup analyses were performed for PSA-DT ≤ 6 months and 6–10 months. Secondary outcomes were overall grade 3+ AEs and specific grade 3+ AEs, namely fatigue, hypertension, and falls.

### Search strategy, study selection, and data collection

The search strategy was based on previous reports [4]. PubMed was searched to identify all available reports addressing combination therapy in high-risk nmCRPC, published before 1 October 2020 (Fig. 1). References from editorials, conference publications, commentaries, review articles, as well as from the three included studies were hand searched and cross-referenced to ensure completeness.

### Study review methodology and risk of bias assessment

Two independent reviewers (MW and LN) performed an initial screening of the existing literature about combination therapy in high-risk nmCRPC, according to PRISMA assessment and in agreement with previous methodology [4, 13, 14]. The relevance of the publication/abstract was confirmed/denied after data extraction and critical review. Disagreements were resolved via consensus with the senior author (PIK).

The “risk-of-bias” evaluation of each study was assessed according to The Cochrane Collaboration’s tool for risk of bias assessing [15]. This tool assesses selection bias (random sequence generation and allocation concealment), reporting bias, performance bias, attrition bias, detection bias, and other sources of bias (Supplementary Fig. 1).

### Statistical analysis

Median OS was defined as the median time from initiation of ADT or combination therapy until the patient’s death or censoring. For OS outcome, we conducted an NMA using random models with a Bayesian approach for direct and indirect treatment comparisons with ADT and alternative treatments [16, 17]. In the assessment of OS, contrast-based analyses were applied with estimated differences in the log hazard ratio (HR) and the standard error calculated from the published HRs and confidence intervals (CI) [18]. The relative treatment effects were presented as HRs and 95% credible interval (CrI). For the assessment of all AE comparisons, arm-based analyses were performed to estimate the median difference (MD) of all shown AEs (and 95% CrI) from the available data presented in the three selected manuscripts or supplemental materials. In addition, we estimated the relative ranking of different treatments for each outcome by using the P-score, according to previous methodology [4, 19, 20]. All analyses were performed with R software environment (version 3.4.3, R Project for Statistical Computing, www.r-project.org) for statistical computing and graphics. Statistical significance was set at *p* < 0.05 [21].

## Results

### Descriptive characteristics of the included studies

The search methodology yielded RCT with updates that were prompted by more mature follow-up [1–3]. In all three RCTs, the inclusion criteria consisted of nmCRPC with PSA-DT < 10 months and an absolute baseline PSA > 2 ng/ml. Patients with regional lymph node metastases were only allowed in the SPARTAN and ARAMIS trials and their proportions differed (Table 1). In the SPARTAN trial 16 and 16% harbored regional lymph node metastases, in respective control and treatment arms vs. 12 and 10% respectively in the ARAMIS trial.

**Table 1:** Table summarizing randomized controlled studies addressing non-metastatic castration-resistant prostate cancer (nmCRPC) patients.

Study/molecule	SPARTAN/apalutamide	PROSPER/enzalutamide	ARAMIS/darolutamide
Author, Year	Smith et al., 2020 [1]	Sternberg et al., 2020 [2]	Fizazi et al., 2020 [3]
Inclusion criteria	nmCRPC, N0/N1, PSA-DT < 10 months, PSA > 2 ng/ml	nmCRPC, N0, PSA-DT < 10 months, PSA > 2 ng/ml	nmCRPC, N0/N1, PSA-DT < 10 months, PSA > 2 ng/ml
Number total (control/treatment)	1207 (401/806)	1401 (468/933)	1509 (554/955)
Median follow-up	52 months	48 months	28 months
Median age (range) (control vs. treatment)	74 (52–97) vs. 74 (48–94)	73 (53–92) vs. 74 (50–95)	74 (50–92) vs. 74 (48–95)
Median PSA at baseline (control vs. treatment)	7.96 vs. 7.78 ng/ml	10.2 vs. 11.1 ng/ml	9.7 vs. 9.0 ng/ml
Median PSA-DT (control vs. treatment)	4.5 vs. 4.4 months	3.6 vs. 3.8 months	4.7 vs. 4.4 months
Proportion PSA-DT ≤ 6 months (control vs. treatment)	70.8 vs. 71.5%	77 vs. 77%^a^	67 vs. 70%
Gleason score 8–10 (control vs. treatment)	43.7 vs. 43.5%	44.2 vs. 40.8%	NA
RP or RT (control vs. treatment)	76.6 vs. 76.6%	NA	NA
The proportion of N1 (control vs. treatment)	16.2% vs. 16.5%	0% vs. 0%	12% vs. 10%
Overall survival (control vs. treatment)	59.9 (52.8–NR) vs. 73.9 (61.2–NR); HR: 0.79, CI: 0.65–0.96	56.3 (54.5–63.0) vs. 67.0 (64.0–NR); HR: 0.73, CI: 0.61–0.89	NR vs. NR; HR: 0.69, CI: 0.53–0.88
Three-year overall survival rate(control vs. treatment)^b^	78 vs. 82%	75% vs. 82%	77 vs. 84%
Overall survival for PSA-DT ≤ 6 months(control vs. treatment)	58.7 vs. 61.3 months; HR: 0.84, CI: 0.68–1.05	NA vs. NA; HR: 0.69, CI: 0.56–0.86^a^	NA vs. NA; HR: 0.74, CI: 0.55–0.99
Overall survival for PSA-DT > 6 months (control vs. treatment)	61.7 vs. NR months; HR: 0.65, CI: 0.44–0.97	NA vs. NA; HR: 0.90, CI: 0.59–1.36	NA vs. NA; HR: 0.55, CI: 0.35–0.88
Metastatic progression-free survival (control vs. treatment)	16.2 vs. 40.5; HR: 0.30, CI: 0.24–0.36	14.7 (14.2–15.0) vs. 36.6 (33.1–NR); HR: 0.29, CI: 0.24–0.35	18.4 (15.5–22.3) vs. 40.4 (34.3–NR);HR: 0.41, CI: 0.34–0.50
Any AE (control vs. treatment)	94.0 vs. 97.0%	82.0 vs. 94.0%	79.2 vs. 85.7%
AE ≥ grade 3 (control vs. treatment)	36.5 vs. 59.0%	27.0 vs. 48.0%	25.1 vs. 30.3%
AE: Fatigue grade 3–4 (control vs. treatment)	0.3 vs. 0.9 %	1.0 vs. 4.0%^c^	0.9 vs. 0.4%
AE: Hypertension grade 3–4 (control vs. treatment)	12.0 vs. 16.0%	2.0 vs. 6.0%^c^	2.3 vs. 3.5%
AE: Fall grade 3–4 (control vs. treatment)	0.8 vs. 2.7%	1.0 vs. 2.0%^c^	0.7 vs. 0.9%

*ADT* androgen deprivation therapy, *PSA* prostate-specific antigen, *PSA-DT* prostate-specific antigen doubling time, *RP* radical prostatectomy, *RT* radiotherapy, *NR* not reached, *HR* hazard ratio, *CI* confidence interval, *AE* adverse event.

^a^Reported rates for PSA-DT <6 months.

^b^Estimated from overall survival curves.

^c^Reported rates for AEs grade ≥3.

The combined population of the three trials consisted of 4117 patients. The sample sizes of treatment arms ranged from 806 to 955 vs. 401 to 554 for the control arms (ADT). It is noteworthy that the median age, median baseline PSA and median PSA-DT were virtually identical within all three RCTs and ranged from 73 to 74 years, 7.8 to 11.1 ng/ml, and 3.6 to 4.7 months, respectively. Similarly, the proportions of PSA-DT ≤ 6 patients were also virtually identical within all three RCTs and ranged from 67 to 77%. Finally, within two studies, that reported Gleason Score, the proportions of scores 8–10 were also virtually the same and ranged from 40.8 to 44.2% (SPARTAN and PROSPER trial). Conversely, the median follow-up durations within the three studies differed: SPARTAN 52 months, PROSPER 48 months, and ARAMIS 28 months.

### Network meta-analysis: overall survival

**Fig. 2:**
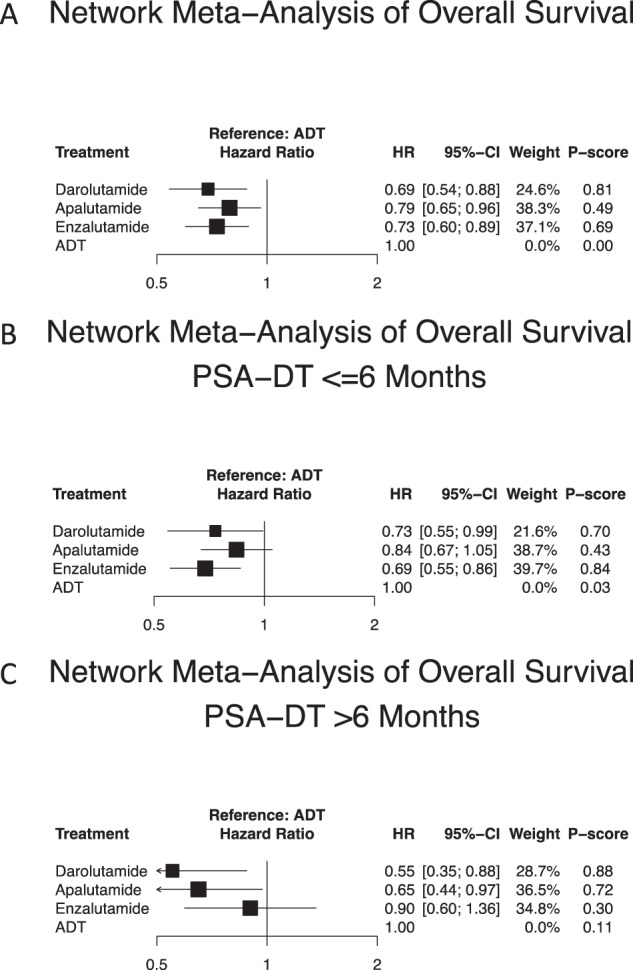
Forest plots of network meta-analysis regarding survival. Forest plots of network meta-analysis depicting the association of systemic therapy in non-metastatic castration-resistant prostate cancer patients for (**A**) overall survival and (**B**) overall survival in patients with PSA doubling time (PSA-DT) ≤ 6 months and (**C**) overall survival in patients with PSA-DT > 6 months. ADT androgen deprivation therapy, HR hazard ratio, CI confidence interval.

Relative to ADT (Fig. 2A), all three ARATs provided longer OS, according to predefined statistical criteria. Specifically, apalutamide, enzalutamide and darolutamide yielded HRs (CrI) of respectively 0.79 (0.65–0.96), 0.73 (0.60–0.89) and 0.69 (0.54–0.88). Based on NMA-derived ranking quantifying the highest likelihood of providing maximal OS benefit, darolutamide ranked first (P-score: 0.81) and was followed by enzalutamide (P-score: 0.69) and more distantly by apalutamide (P-score: 0.49), in that order.

### Network meta-analysis: overall survival in PSA-DT ≤ 6 months patients

Relative to ADT (Fig. 2B), only two out of three ARATs (enzalutamide and darolutamide) exhibited a statistically significant OS benefit in patients with PSA-DT ≤ 6 months, according to predefined criteria. The specific HRs (CrI) were respectively 0.69 (0.55–0.86) for enzalutamide and 0.73 (0.55–0.99) for darolutamide. The exception consisted of apalutamide (HR: 0.84, CrI: 0.67–1.05). Based on NMA-derived ranking quantifying the highest likelihood of providing maximal OS benefit in patients with PSA-DT ≤ 6 months, enzalutamide ranked first (P-score: 0.84), followed by darolutamide (P-score: 0.70).

### Network meta-analysis: Overall survival in PSA-DT 6–10 months patients

Relative to ADT (Fig. 2B), only two out of three ARATs (apalutamide and darolutamide) exhibited a statistically significant OS benefit in patients with PSA-DT 6–10 months, according to predefined criteria. The specific HRs (CrI) were respectively 0.65 (0.44–0.97) for apalutamide and 0.55 (0.35–0.88) for darolutamide. The exception consisted of enzalutamide (HR: 0.90, CrI: 0.60–1.36). Based on NMA-derived ranking quantifying the highest likelihood of providing maximal OS benefit in patients with PSA-DT 6–10 months, darolutamide ranked first (P-score: 0.88), followed by apalutamide (P-score: 0.72).

### Network meta-analysis: grade 3+ AEs

**Fig. 3:**
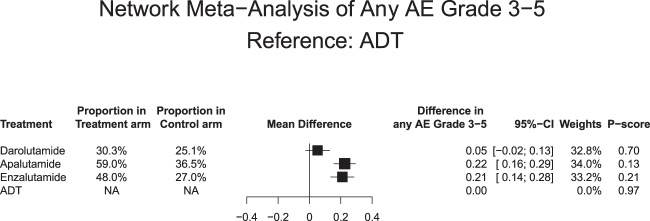
Forest plots of network meta-analysis regarding AEs. Forest plots of network meta-analysis depicting the association of systemic therapy in non-metastatic castration-resistant prostate cancer patients for any grade 3–5 adverse event (AE). ADT androgen deprivation therapy, CI confidence interval, NA Not applicable.

Relative to ADT (Fig. 3), two out of three ARATs (apalutamide: MD +22%, CrI: +16 to +29% and enzalutamide: MD +21%, CrI: +14 to +28%) exhibited a statistically significantly higher likelihood of grade 3+ AEs. The exception consisted of darolutamide. Its rate of grade 3+ AEs did not differ from ADT (MD: +5%, CrI: −2 to +13%). Based on NMA-derived ranking quantifying the lowest likelihood of grade 3+ AEs, darolutamide ranked first (P-score: 0.70) and was very distantly followed by enzalutamide (P-score: 0.21) and apalutamide (P-score: 0.13), in that order.

### Network meta-analysis: grade 3–4 fatigue, hypertension, and falls

**Fig. 4:**
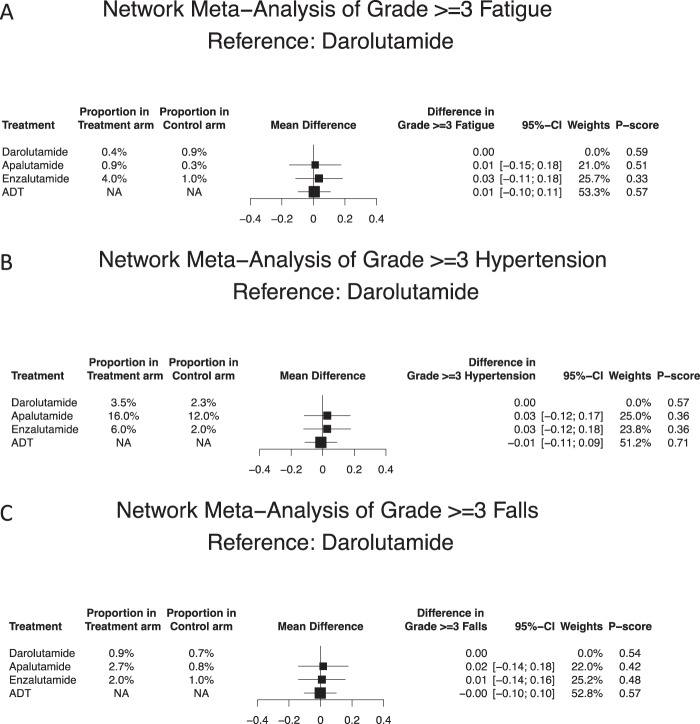
Forest plots of network meta-analysis regarding specific AEs. Forest plots of network meta-analysis depicting the association of systemic therapy in non-metastatic castration-resistant prostate cancer patients for specific adverse events (AE) (**A**) fatigue grade ≥3 and (**B**) hypertension grade ≥3 and (**C**) fall grade ≥3. ADT androgen deprivation therapy, CI confidence interval, NA not applicable.

Regarding grade 3–4 fatigue and relative to darolutamide, apalutamide and enzalutamide were not significantly different (Fig. 4A). Based on NMA-derived ranking quantifying the lowest likelihood of grade 3–4 fatigue, darolutamide ranked first (P-score: 0.59), followed by apalutamide (P-score: 0.51) and enzalutamide (P score: 0.33), in that order.

Regarding grade 3–4 hypertension and relative to darolutamide, apalutamide and enzalutamide were not significantly different (Fig. 4B). Based on NMA-derived ranking quantifying the lowest likelihood of 3–4 grade hypertension, darolutamide ranked first (P score: 0.57), followed by apalutamide (P score: 0.36) and enzalutamide (P score: 0.36).

Regarding grade 3–4 falls and relative to darolutamide, apalutamide and enzalutamide were not significantly different (Fig. 4C). Based on NMA-derived ranking quantifying the lowest likelihood of 3–4 grade falls, darolutamide ranked first (P score: 0.54), followed by enzalutamide (P score: 0.48) and apalutamide (P score: 0.42), in that order.

## Conclusion

We conducted a systematic review and completed an NMA that addressed the effect of three ARATs on OS and grade 3+ AEs in high-risk nmCRPC patients, relative to ADT. Three-phase III RCTs qualified for inclusion based on proven OS benefit, relative to ADT, according to predefined criteria. Several noteworthy findings were made.

First, there are three ARATs that demonstrated an OS benefit, relative to ADT in high-risk nmCRPC within three RCTs. The findings of these three RCTs have been repeatedly updated with the availability of progressively longer follow-up. Despite the current availability of most mature follow-up, direct or indirect OS comparisons using the most mature reports have not been made. This unmet need represented the rationale for the current study and the NMA. Based on the absence of direct comparisons between the three ARATs and as endorsed by the Cochrane Collaboration, we designed an NMA with the intent of providing a rank order with respect to cancer-control and toxicity outcomes of the three ARATs based on most mature observations [1–3, 22]. Such methodology was previously used on five occasions. All previous NMAs relied on less mature data analyses [6–8]. Moreover, one NMA also included studies without proven OS benefit [5]. Finally, the NMA reported by Mori et al. relied on the most mature enzalutamide data, but not on most mature apalutamide and enzalutamide reports [4]. In consequence, the current NMA is clearly justified and fills a void.

Second, the results of our NMA demonstrated differences in OS, when the three ARATs were indirectly compared. Specifically, darolutamide ranked first (P score: 0.81), followed by enzalutamide (P score 0.69) and apalutamide (P score 0.49). Invariably, the NMA-derived P scores corresponded to the strength of the OS effect size, evidenced by HRs that respectively ranged from 0.69 to 0.79. The current observations that are based on most mature follow-up disagree with reports of Hird et al. and Mori et al. regarding efficacy, where instead of darolutamide ranking first according to the current data, apalutamide ranked first but was nonetheless closely followed by darolutamide [4, 7]. The difference between the current NMA and the previous NMAs emphasizes the importance of interpreting the most mature data that provide the most definitive and robust efficacy estimates. The existence of a rank order according to the current NMA findings further validates an uncertainty about the therapeutic equivalence of the three ARATs, as was already shown in previous studies [4, 7]. Unfortunately, the uncertainty about potential differences in efficacy can only be resolved with non-inferiority trials. Such trials are unlikely to ever be completed.

Third, unlike previous NMAs, we stratified our observations regarding OS benefit according to PSA-DT. In the subgroup with PSA-DT ≤ 6 months, NMA rank order addressing OS identified enzalutamide as first, darolutamide as second, and apalutamide as third with respect to their efficacy. This order was different from the rank order recorded in the overall analysis without PSA-DT stratification, where darolutamide was most efficacious and was followed by enzalutamide and apalutamide. Conversely, in the subgroup with PSA-DT 6–10 months, NMA rank order addressing OS identified darolutamide as first, apalutamide as second, and enzalutamide as third, with respect to their efficacy and perfectly, correspond to the rank order recorded in the overall analysis. The above results also perfectly corresponded to associated HRs from the original phase III RCTs. To the best of our knowledge, we are the first to report on subgroup analyses according to PSA-DT. The observed rank order in both subgroups is based on final OS data. Finally, as for the overall data, the existence of a rank order that is different from the overall rank order adds to the uncertainty about potentially efficacy differences. Those uncertainties ideally would require prospective non-inferiority trials to achieve the final resolution.

Fourth, although previous NMAs reported comparisons of 3+ grade AEs, we reassessed those comparisons using the most mature follow-up. Regarding lowest overall grade 3+ AEs, darolutamide ranked first, followed by enzalutamide and apalutamide. These findings are consistent with previous NMA findings, despite less mature follow-up [4, 6]. Our NMA did not focus on overall AEs of all grades, unlike Kumar et al. and Mori et al. The decision to focus on grade 3+ AEs was based on the observation that virtually all patients in all three trials (97% in SPARTAN, 94% in PROSPER and 86% in ARAMIS) exhibit at least one AE during follow-up [1, 4, 6]. In consequence, in the context of high-risk nmCRPC treated with the tree ARATs, grade 3+ AEs are of greatest interest for clinicians in treatment decision-making. The observed advantage of darolutamide over enzalutamide and apalutamide with respect to grade 3+ AEs might be explained by its lower penetration of the blood-brain barrier, relative to the two other ARATs and to fewer interactions with other pharmacological agents due to lack of CYP-pathway mediated effects [23–26].

Fifth, we assessed the effect of the three ARATs according to specific grade 3–4 AEs, namely fatigue, hypertension, and falls, relative to ADT. The resulting findings according to NMA rank order identified darolutamide as the ideal treatment option due to its lowest likelihood of any of the three addressed grade 3–4 specific AEs. Those findings are particularly noteworthy since all three examined grade 3–4 AEs are important and may result in temporary or permanent ARAT discontinuation. Other grades 3–4 AEs are also important. However, the rank order of the three ARATs could not be examined in their regard, due to data unavailability. Similarly, we could not address the lesser grade of some important AEs, such as for example grade 2 fatigue, due to data unavailability.

Finally, although the five main observations made using the NMA-based approach provide an appealing rank order with respect to OS and toxicity, this rank order should be interpreted with caution. The latter is required based on important differences between the three examined RCTs with respect to their design, patient characteristics of the control and treatment groups, as well as their maturity. Indeed, study maturity differed extensively between the three trials due to the median follow-up duration that ranged from 28 to 52 months. Especially, the darolutamide trial is limited by less mature data and median OS could not be reached for the ADT and darolutamide group. Added maturity may change the observed relationships between the three ARATs, as was observed when the current findings were compared to those of Mori et al., with less mature data [4]. Moreover, added maturity may change the HRs of the included studies as was observed in several previous studies [27–31]. In addition, differences in data maturity and duration of follow-up may affect cumulative rates of AEs. Longer follow-up invariably will result in a higher rate of toxicities. In consequence, darolutamide data that provide the most favorable toxicity profile may worsen, when median follow-up duration is extended from the current 28 months to longer follow-up, as is the case for apalutamide and enzalutamide. To which extent differences in molecular structure between the three compared ARATs and their activity on the androgen receptor explain the current findings, is not clear. However, since darolutamide’s penetration of the brain-blood-barrier is low, lower rates of central nervous system AEs may be explained by this hypothesis [32].

Differences in patient characteristics that exist between the three-phase III RCTs are also important to consider in the interpretation of the current, as well as all previous NMAs. Very similar distribution of PSA, PSA-DT, baseline Gleason scores and regional lymph node metastases most likely had marginal if any contribution to population heterogeneity, within the three- phase III RCTs. However, study designs differed with respect to PSA- DT definitions. Specifically, the PROSPER trial relied on PSA-DT of less than 6 months. Conversely, the SPARTAN and ARAMIS trials included patients with PSA-DT for up to six months. Such difference may be marginal. However, it requires mention. In addition, all studies relied on conventional imaging. Although the use of conventional imaging did not differ between studies, it is of importance to emphasize that patient inclusion in the category of high-risk nmCRPC was much higher than if PSMA PET/CT was systematically obtained. Moreover, the timing of AE capture and their definitions may have also demonstrated small, albeit potentially important differences that influenced AE rates of the three ARATs. However, it is unlikely that study design differences have induced important confounding variables that prevent valid direct or indirect comparisons between the three RCTs since the endpoint of interest corresponds to OS. In all three RCTs, the assessment of this endpoint is the same. In addition, differences with respect to patterns of PSA-progression-free survival and metastatic progression-free survival (Supplemental Fig. 2) exist between the three RCTs. All of the above potential differences, regardless of their marginal or more important magnitude, were not and could not be formally addressed or adjusted for within the NMA methodology.

Moreover, study designs differed with respect to PSA-DT definitions. Specifically, the PROSPER trial relied on PSA-DT of less than 6 months. Conversely, the SPARTAN and ARAMIS trials included patients with PSA-DT for up to six months. Such difference may be marginal. However, it requires mention. In addition, all studies relied on conventional imaging. Although the use of conventional imaging did not differ between studies, it is of importance to emphasize that patient inclusion in the category of high-risk nmCRPC was much higher than if PSMA PET/CT was systematically obtained. Moreover, the timing of AE capture and their definitions may have also demonstrated small, albeit potentially important differences that influenced AE rates of the three ARATs. However, it is unlikely that study design differences have induced important confounding variables that prevent valid direct or indirect comparisons between the three RCTs since the endpoint of interest corresponds to OS. In all three RCTs, the assessment of this endpoint is the same. In addition, differences with respect to patterns of PSA-progression-free survival and metastatic progression-free survival (Supplemental Fig. 2) exist between the three RCTs. All of the above potential differences, regardless of their marginal or more important magnitude, were not and could not be formally addressed or adjusted for within the NMA methodology.

Taken together, the current NMA provides the most mature, definitive, and robust comparisons of OS benefits from darolutamide, enzalutamide and apalutamide, relative to ADT in high-risk nmCRPC. Second, unlike previous NMA reports that were based on less mature comparisons, the current NMA ranked darolutamide first regarding efficacy, followed by enzalutamide and apalutamide, in that order. Third, we are the first to report subgroup analyses of efficacy with respect to OS. The PSA-DT ≤ 6 months subgroup revealed the highest NMA-based efficacy for enzalutamide, which was followed by darolutamide and apalutamide. This rank order differed from the overall rank order that was defined without stratifying for baseline PSA-DT. It is of note that in PSA-DT 6–10 months subgroup the same rank order was recorded, as in the entire cohort. Finally, regarding grade 3+ AEs, darolutamide invariably was ranked as the ideal treatment option. However, its rank may at least partially be related to the shortest follow-up that was available to observe grade 3+ AEs, relative to the other two ARATs. All of the above observations require consideration of heterogeneity regarding patient characteristics, maturity, and study design when the current study is interpreted.

## Supplementary information

Supplemental Figure 1

Supplemental Table 2

## Data Availability

Code will be made available for bona fide researchers on request.
