# Correction: Overall survival and adverse events after treatment with darolutamide vs. apalutamide vs. enzalutamide for high-risk non-metastatic castration-resistant prostate cancer: a systematic review and network meta-analysis

**DOI:** 10.1038/s41391-023-00656-4

**Published:** 2023-03-10

**Authors:** Mike Wenzel, Luigi Nocera, Claudia Collà Ruvolo, Christoph Würnschimmel, Zhe Tian, Shahrokh F. Shariat, Fred Saad, Derya Tilki, Markus Graefen, Luis A. Kluth, Alberto Briganti, Philipp Mandel, Francesco Montorsi, Felix K. H. Chun, Pierre I. Karakiewicz

**Affiliations:** 1 Department of Urology, Goethe University Hospital Frankfurt, Frankfurt am Main, Germany; 2 Division of Urology, Cancer Prognostics and Health Outcomes Unit, University of Montréal Health Center, Montréal, QC Canada; 3 Department of Urology and Division of Experimental Oncology, URI, Urological Research Institute, IRCCS San Raffaele Scientific Institute, Milan, Italy; 4 Department of Neurosciences, Reproductive Sciences and Odontostomatology, University of Naples Federico II, Naples, Italy; 5 Martini-Klinik Prostate Cancer Center, University Hospital Hamburg-Eppendorf, Hamburg, Germany; 6 Department of Urology, Comprehensive Cancer Center, Medical University of Vienna, Vienna, Austria; 7 Department of Urology, Weill Cornell Medical College, New York, NY USA; 8 Department of Urology, University of Texas Southwestern, Dallas, TX USA; 9 Department of Urology, Second Faculty of Medicine, Charles University, Prag, Czechia; 10 Institute for Urology and Reproductive Health, I.M. Sechenov First Moscow State Medical University, Moscow, Russia; 11 Division of Urology, Department of Special Surgery, Jordan University Hospital, The University of Jordan, Amman, Jordan; 12 Department of Urology, University Hospital Hamburg-Eppendorf, Hamburg, Germany

**Keywords:** Cancer therapy, Prostate cancer

Correction to: *Prostate Cancer and Prostatic Diseases* 10.1038/s41391-021-00395-4, published online 30 May 2021

The author wants to make the following corrections:

**Original:**

Table 1: “The proportion of N1 (control vs. treatment) … 29% vs. 17%”

Text pages 140–141: “Patients with regional lymph node metastases were only allowed in the SPARTAN and ARAMIS trials and their proportions differed (Table 1). In the SPARTAN trial 16 and 16% harbored regional lymph node metastases, in respective control and treatment arms vs. 29 and 17% respectively in the ARAMIS trial.”

**Corrected:**

Table 1: “The proportion of N1 (control vs. treatment) … 12% vs. 10%”

Text pages 140–141: “Patients with regional lymph node metastases were only allowed in the SPARTAN and ARAMIS trials and their proportions differed (Table 1). In the SPARTAN trial 16 and 16% harbored regional lymph node metastases, in respective control and treatment arms vs. 12 and 10% respectively in the ARAMIS trial.”

**Original:**

Text pages 145–146: “However, more pronounced differences were recorded in rates of regional lymph node metastases and the study findings need to be interpreted in their light. However, it should also be noted that the distribution of lymph node metastases was virtually the same in the treatment and control arms in the SPARTAN and PROSPER trials. Although the distribution of lymph node metastases was unequally divided in the ARAMIS trial (control: 29 vs. treatment: 17%), a higher prevalence of lymph node metastases in the control arm certainly did not prevent darolutamide from ranking first in overall efficacy analyses. In consequence, it is not clearly evident that lymph node metastases rate differences resulted in important heterogeneity that confounded the study findings.”

**Corrected:**

“Differences in patient characteristics that exist between the three-phase III RCTs are also important to consider in the interpretation of the current, as well as all previous NMAs. Very similar distribution of PSA, PSA-DT, baseline Gleason scores and regional lymph node metastases most likely had marginal if any contribution to population heterogeneity, within the three- phase III RCTs. However, study designs differed with respect to PSA- DT definitions. Specifically, the PROSPER trial relied on PSA-DT of less than 6 months. Conversely, the SPARTAN and ARAMIS trials included patients with PSA-DT for up to six months. Such difference may be marginal. However, it requires mention. In addition, all studies relied on conventional imaging. Although the use of conventional imaging did not differ between studies, it is of importance to emphasize that patient inclusion in the category of high-risk nmCRPC was much higher than if PSMA PET/CT was systematically obtained. Moreover, the timing of AE capture and their definitions may have also demonstrated small, albeit potentially important differences that influenced AE rates of the three ARATs. However, it is unlikely that study design differences have induced important confounding variables that prevent valid direct or indirect comparisons between the three RCTs since the endpoint of interest corresponds to OS. In all three RCTs, the assessment of this endpoint is the same. In addition, differences with respect to patterns of PSA-progression-free survival and metastatic progression-free survival (Supplemental Fig. 2) exist between the three RCTs. All of the above potential differences, regardless of their marginal or more important magnitude, were not and could not be formally addressed or adjusted for within the NMA methodology.”

The original article has been corrected.

