# Supplementary material for: Overall survival and adverse events after treatment with darolutamide vs. apalutamide vs. enzalutamide for high-risk non-metastatic castration-resistant prostate cancer: a systematic review and network meta-analysis
Source: Prostate Cancer Prostatic Dis. 2021 May 30;25(2):139–48. doi: 10.1038/s41391-021-00395-4 (PMC9184262; doi:10.1038/s41391-021-00395-4)
Supplement: Supplementary file 1 — Supplemental Figure 1 [file 41391_2021_395_MOESM1_ESM.docx]

**Supplemental Figure 1.** Risk of bias summary for the three included studies within the current network meta-analysis of high-risk non-metastatic castration-resistant prostate cancer. Green circles represent low risk of bias and confounding, red circles represent high risk of bias and confounding, and yellow circles represent unclear risk of bias and confounding

1. Random sequence generation (selection bias)
2. Allocation concealment (selection bias)
3. Selective reporting (reporting bias)
4. Blinding of participants and personal (performance bias)
5. Incomplete outcome data (attrition bias)
6. Blinding of outcome assessment (detection bias)
7. Other bias
