# Supplementary material for: Overall survival and adverse events after treatment with darolutamide vs. apalutamide vs. enzalutamide for high-risk non-metastatic castration-resistant prostate cancer: a systematic review and network meta-analysis
Source: Prostate Cancer Prostatic Dis. 2021 May 30;25(2):139–48. doi: 10.1038/s41391-021-00395-4 (PMC9184262; doi:10.1038/s41391-021-00395-4)
Supplement: Supplementary file 2 — Supplemental Table 2 [file 41391_2021_395_MOESM2_ESM.docx]

**Supplemental Figure 2.** Median metastatic-free survival (MFS) according to ADT and apalutamide, enzalutamide and darolutamide in high-risk non-metastatic castration resistant prostate cancer.
